# Condom Access in South African Schools: Law, Policy, and Practice

**DOI:** 10.1371/journal.pmed.1000006

**Published:** 2009-01-20

**Authors:** Juliana Han, Michael L Bennish

## Abstract

Juliana Han and Michael L. Bennish discuss their experience developing a policy on condom distribution for Mpilonhle, a South African nongovernmental organization involved in HIV prevention in schools.

In 2007, South Africa's new Children's Act came into effect [1,2], expanding the scope of several existing children's rights and explicitly granting new ones. The Act gives to children 12 years and older a host of rights relating to reproductive health, including access to contraceptives and to information on sexuality and reproduction, and the right of consent to HIV/AIDS testing and treatment [1].

These rights reflect growing concern over the need to prevent HIV in the country's youth. South Africa has the highest number of persons living with HIV in the world [3]. Persons aged 15–24 account for 34% of all new HIV infections and have an HIV prevalence of 10.3% [4,5].

A critical challenge for HIV prevention efforts in adolescents is to ensure that these newly guaranteed reproductive health rights are realized. For youths in South Africa, access to condoms is limited. Barriers to access include substantial travel time and cost of travel to sites of condom distribution [6], the fact that government clinics distributing free condoms are usually closed when students are out of school, the judgmental and often hostile attitude of providers, and the cost of condoms in shops [7].

One way to increase condom access for adolescents is to make condoms available in schools. This is a socially divisive approach. Some believe availability of contraception will encourage sexual activity [8]. Others cite the early age of sexual debut [9,10] and the futility of HIV prevention education that emphasizes condom use without providing sexually active youth with access to condoms [11].

**Summary Points**

- South Africa's recently adopted Children's Act provides children the right to access reproductive health services as a way of addressing the HIV pandemic, but there remains confusion about how socially divisive rights provided for by the Act, such as condom access for youth, will be achieved.
- The Children's Act, together with South African government policies, allows individual schools to decide whether to distribute condoms, but most school staff are unaware of South African policy and regulations governing condom provision in schools.
- Because of confusing and contradictory government policies and public pronouncements regarding provision of condoms in public schools, few schools have undertaken to provide condoms, leaving students, especially in rural areas, with few options for obtaining them.
- PEPFAR regulations potentially conflict with South African law by prohibiting PEPFAR-funded organizations from distributing condoms in schools or providing condom information to youth aged 14 and under.
- The current South African government's policy of leaving the decision of whether to distribute condoms in schools to the School Governing Body of individual schools, rather than enacting a clear national policy, is unlikely to be an effective public health strategy for improving access to condoms for the population of youths at high risk for HIV.

Reflecting these tensions, South African government policy is unclear, and school staff are often unsure if condom distribution in schools is permissible. As a result, most schools hesitate to distribute condoms, and those few that do distribute condoms do so discreetly [12].

Given the continuing high HIV incidence rates in youth, it is important to examine current South African laws and policies governing condom distribution in schools, policies of international donor agencies supporting HIV/AIDS prevention programs in South Africa [13], and community perceptions surrounding condom distribution in schools. We reflect on our experience in developing a policy on condom distribution for Mpilonhle, a nongovernmental organization involved in HIV prevention in schools, focusing on germane South African and PEPFAR (the US President's Emergency Plan for AIDS Relief) policies and on the attitudes of students, school staff, and parents towards condom distribution in schools.

## Law and Policy

### South African laws and policies.

The overarching legal document governing children's rights to access contraceptives is the Children's Act No. 38 of 2005 (“Children's Act”). The sections of the Children's Act regarding the responsibilities of the national government, such as reproductive health rights and children's courts, were approved by the President in 2006 [1]. Provisions regarding the responsibilities of provincial governments, such as foster care and child-care centers, are contained in the Children's Amendment Act, approved by the President in 2008 [14].

The Children's Act delineates rights not present in the Child Care Act of 1983, many of which are relevant to youth health programs. For instance, every child, regardless of age, has the right to “have access to information on . . . the prevention and treatment of ill-health and disease, sexuality and reproduction” [15]. A 12-year-old child can consent to HIV testing [16], and children under 12 years can also consent if they are of sufficient maturity to understand the benefits, risks, and social implications of a test.

The Children's Act states that no person may refuse to sell condoms to a child 12 years or older, or refuse to provide such a child with condoms on request where such condoms are distributed free of charge [17]. No further regulations are needed to effect these rights [2]. However, whether these rights are appropriate remains the focus of intense debate [18,19].

In addition to the Children's Act, the South African Department of Education (DOE) policies also govern condom distribution in schools. The current DOE policy is a politically pragmatic solution to the national debate: let local schools decide for themselves. In a 1999 policy document (still in force) on HIV and AIDS in public schools, the DOE stated that each school can decide “whether condoms need to be made accessible within a school . . . and if so under what circumstances” [20].

This decentralization reflects the mechanics of policy implementation. The national Minister of Education determines national policy, which drives policy and implementation at the provincial level [21]. Policy updates then trickle down through regional DOE offices, which pass them to local schools. The ultimate governance of every public school is vested in its School Governing Body (SGB), consisting of parents, school staff, and students [22].

In the case of condom distribution in schools, the policy of decentralization has been poorly communicated. Most school staff are unaware of any policies on condom distribution in schools. Perhaps more worrisome, many funding agencies, advocacy groups, and government officials believe that condom distribution in schools is impermissible as a matter of stated policy. This view seems based on statements by senior government officials, including the Minister of Education, suggesting that condom distribution in schools is inappropriate [12,23,24]. Statements to the press, no matter how public, are not official policy. Education policy must be made according to defined procedures, including notice and publication in the Government Gazette [21].

The Children's Act thus preserves the schools' right to choose to distribute condoms, with one modification. If schools do distribute condoms, they must provide them to all students 12 and over. The Act does not impose an obligation on the government to distribute condoms. The condom access clause is a “negative right,” which obligates the government to refrain from certain actions. It is not a “positive right,” such as the Constitutional right that obligates the government to provide access to health care services [25]. The latter right was the basis for the South African Constitutional Court's decision that drugs to prevent mother-to-child transmission of HIV must be made available to all HIV-infected women giving birth in state health facilities [26].

### Policies of funding agencies.

Restrictions of funding agencies, other than the South African government, that support HIV prevention efforts are also relevant to condom access for adolescents. The United States government is the largest bilateral donor to South Africa's health sector [27]. Through PEPFAR, it allocated US$398 million in US fiscal year 2007 for South African HIV/AIDS programs [28].

PEPFAR's emphasis on abstinence in prevention programs limits condom interventions [29]. PEPFAR global guidelines prohibit use of plan funds for the physical distribution of condoms in schools or the provision of condom information to youth aged 14 and under [29].

This policy conflicts with South African law. It is unclear how health workers supported by PEPFAR who work in South African institutions, such as schools, should act if youngsters under 14 but above 12 years request information or condoms. The Children's Act grants all children the right to access health information and children 12 and older the right to access condoms, but the workers are ostensibly constrained by PEPFAR policies. That PEPFAR's policies may hinder the provision of beneficial condom programs for youth has been noted [30].

## Community Attitudes and Responses

### Discussions with the community on program development and implementation.

To determine how Mpilonhle should, as part of its programs, approach the provision of condoms in schools, we spoke to persons in the communities of four high schools in which Mpilonhle intended to provide services. The schools are located in rural northern KwaZulu-Natal, the province with the highest prevalence of HIV [4]. These schools have student enrollment of 400–900 students aged 12–22 years, and instruction is in English.

Discussions were held at one SGB meeting, which included the principal, one teacher, six parents, and one student representative, and at two parent meetings, each with approximately 100 adults, primarily women, in attendance. Individual interviews were conducted with principals and staff at each of the four schools, including teachers of the Life Orientation subject, which includes HIV/AIDS prevention. At three schools, discussions were held with groups of five to ten students of both sexes from grades 8–12, selected for participation by school staff.

All meetings began with structured questions, including: (1) how schools are addressing HIV/AIDS prevention, including condom education or distribution; (2) how the respondents view condom distribution in schools; and (3) what specific strategies Mpilonhle could use to increase the efficacy of condom distribution in schools.

Students were asked about condom availability and use. School administrators were asked about their awareness of national policy on condoms in schools and their understanding of education policy implementation.

These questions led to open-ended discussions regarding the impact in schools of HIV/AIDS and other problems, such as teenage pregnancy. Questions were asked in English and, in SGB and parent meetings, translated into Zulu.

These discussions were conducted to enable implementation of a service program. Determining community attitudes was required to ascertain whether Mpilonhle could distribute condoms in the schools in which it intended to work. Because these discussions were conducted for program implementation and not for research purposes, and because no experimentation on humans was involved, institutional review board approval was not obtained.

We do not claim that these responses are representative of South Africa. Rather, these comments were critical in helping Mpilonhle formulate an effective HIV prevention program for local youth. Mpilonhle now provides condoms at the schools it serves in accordance with these findings.

### Community response.

Attitudes about condoms in schools at the community level vary widely. Cultural and moral concerns remain strong among both parents and students, including the preservation of such traditional values as abstinence until marriage. Many parents and some students, but few school staff, felt that condom availability would promote sexual activity and undermine traditional values.

These concerns were balanced by a strong sense of the growing urgency of the HIV/AIDS epidemic. Most people we spoke to indicated that they knew family or friends affected by AIDS and spoke about the emotional impact of burying people from the disease every weekend.

Some community members were also keenly interested in access to condoms to prevent teenage pregnancy, sharing the KwaZulu-Natal government's concern regarding the increase in pregnancies in schools [31]. Others, however, stated that pregnancy prevention will not be a compelling reason for condom use because many adolescent girls want the government's child support grant, even though studies have found no correlation between the grant and teenage fertility [32,33].

### The importance of procedure.

With such diversity of opinion, administrators were not eager to be first movers and insisted on a procedure for ascertaining community support for condom distribution programs. First, schools should consult as many parents and guardians as possible; outside groups such as nongovernmental organizations could help facilitate this discourse. Second, schools should involve the larger community, including traditional (tribal) leadership. Lastly, the SGB should ultimately decide whether to proceed with condom distribution.

Parental support was perceived to be the key factor for program success. Students felt that they could not communicate frankly with their parents about sex. The natural awkwardness between adolescents and parents is reinforced by cultural practices, such as virginity testing, that further stigmatize sexual activity and open discussion [34,35].

Many parents lacked basic knowledge of HIV/AIDS and condoms. Parents questioned the efficacy of condoms and expressed faulty beliefs about HIV transmission, for instance, that a child might get infected by playing with used condoms. Several adults indicated that they themselves did not know how to use a condom. Many parents complained of adults in the community who, accessing condoms in places like shebeens (taverns), would dispose of used condoms indiscriminately. Concerns about wastage and litter were surprisingly common.

School staff also felt it important to consult the greater community, including traditional leaders. This might be accomplished by having a traditional leader present at school meetings. Although the traditional authority has no official role in the operation of schools, condom distribution was seen by some to threaten the moral fiber of the community, areas of concern for traditional leadership [36].

With sufficient parental and community support, school educators, administrators, and governing body members indicated that they would be eager to distribute condoms in schools. Educators also recommended distribution points for school staff; HIV prevalence among teachers in KwaZulu-Natal is estimated at 22% [37].

### Options for condom distribution in schools.

Once a school decides to provide access to condoms, it must decide on the logistics of distribution. The past experiences of two schools in which we work were instructive. At one school, a box of condoms issued by the national Department of Health (DOH) was brought to the school by a nongovernmental organization and placed in the principal's office. In the more than a year that the condoms remained in the office, not a single student approached school authorities to request condoms.

In another school, an educator obtained a box of condoms from a local clinic and placed it in an unlocked cupboard drawer in the school library. Students had to ask for the key to the library, but the educator who had the key said that she never questioned the students' reasons. She reported that the condom box was quickly empty.

Both students and teachers indicated that having an authority figure serve as gatekeeper for the condom supply would deter access in schools, just as it does in clinics. Students fear authority figures discovering that they are sexually active, scolding them for having sex, and asking disapproving questions.

Many students suggested putting unmonitored dispensers in the toilets and classrooms and believed that, with proper education, they would be used appropriately. Other students disagreed, believing that such a setup would encourage litter and misuse—concerns akin to those of their parents. Regardless, if an authority figure is involved, students were adamant that the figure be nonjudgmental. One student described the proper adult role as “control of the condoms, not of the learner.”

## Discussion

Despite the high incidence of HIV in adolescents [38] and the efficacy of condoms in preventing HIV transmission, condom use rates among adolescents remain low, due at least in part to limited access. Especially in rural areas, schools are one of the few sites accessible to large numbers of youth; yet, condom distribution is rarely undertaken in schools.

Although this is a contentious issue, we have found that school staff and students generally support the distribution of condoms in schools but are confused about governmental policy. The national policy, that schools can decide whether condom distribution is beneficial, is one sentence in a national DOE document of which local schools seem unaware. Statements of government officials against condom distribution further obscure actual government policy.

This ambiguity, created by unclear policies and contradictory public statements, has characterized South Africa's approach to other HIV-related issues as well. Both the former Health Minister and the previous President have voiced doubts about the causation and treatment of HIV/AIDS, which at the worst retard national AIDS policy and at the least hinder public understanding of the disease [39].

One rationale for South Africa's decentralized approach to condom distribution in schools is sensitivity to local attitudes. SGBs are arguably in the best position to gauge community views. How exactly to elicit those views, however, is unclear. In parent meetings, men were most outspoken, voicing concerns about the effect of condom distribution on “traditional” values. However, both women and men supported condom distribution more openly in private conversations. School administrators are also unsure what level of consensus would be sufficient to move forward. Should a vote be taken, and if so, should a majority or near unanimity be required? Unfortunately, the latter scenario may always preclude potentially beneficial action.

While decentralization of decision-making on socially divisive issues may be politically expedient, its effectiveness in spurring needed action is questionable. Given the dire risks that still face South African youths, leaving major public health initiatives to local option may be an insufficient governmental response.

The South African government's apparently contradictory actions reflect in part the presence of deeply divergent forces in society. Many in South Africa have supported expanding children's rights to reproductive health services, reflecting the desire in the post-apartheid era to expand individual rights in response not only to injustices of the past, but also to the harsh realities of the present.

The government could be similarly bold in policy implementation and mandate that schools provide condoms. The DOH, which already distributes condoms in public access points [40], could add schools as a distribution site [41]. The DOH, the DOE, and the Department of Social Development (under whose jurisdiction the Children's Act falls) could coordinate more closely, perhaps building on existing programs such as the DOE Life Orientation curriculum and the DOH School Health Policy, to support more effective education on, and access to, condoms.

Together, schools and the government could also increase efforts to educate communities about adolescent condom use. Educators should make clear that provision of condoms is not an endorsement of sexual activity. Empirical research on distribution efforts in South African schools can address fears of increased sexual activity or wastage. At least one study suggests that wastage of condoms distributed from public access points in South Africa is not substantial [42]. In the United States, where social consensus on condoms in schools has also been elusive, studies show that high school distribution of condoms has not resulted in increased sexual activity [43] but has increased condom usage [44].

Community concerns may also be mitigated by tailoring the logistics of condom distribution in schools. The degree of adult supervision over the condom supply may depend on the needed balance between open access and potential misuse.

Access alone is not sufficient. Programs will need to teach proper condom use and address factors contributing to inconsistent use [34], such as a lack of perceived risk of HIV, influence of peer beliefs, and unequal power relations between genders [45,46].

The need to balance sensitivity to local attitudes and urgent national health needs is not unique to the issue of condom access for youths, nor to South Africa, but is part of any policy discussion on socially divisive issues with compelling public health implications. Our experience with South African and PEPFAR policies regarding condom distribution in schools indicates that the present balance disfavors the health of the country's youth and demonstrates a need for clearer and more decisive national action.

## Abbreviations

DOE: Department of Education
DOH: Department of Health
PEPFAR: President's Emergency Plan for AIDS Relief
SGB: School Governing Body
